# Pet Ownership Patterns and Successful Aging Outcomes in Community Dwelling Older Adults

**DOI:** 10.3389/fvets.2020.00293

**Published:** 2020-06-25

**Authors:** Erika Friedmann, Nancy R. Gee, Eleanor M. Simonsick, Stephanie Studenski, Barbara Resnick, Erik Barr, Melissa Kitner-Triolo, Alisha Hackney

**Affiliations:** ^1^Department of Organizational Systems and Adult Health, University of Maryland School of Nursing, Baltimore, MD, United States; ^2^Center for Human Animal Interaction, Department of Psychiatry, School of Medicine, Virginia Commonwealth University, Richmond, VA, United States; ^3^Intramural Research Program, National Institute on Aging, National Institutes of Health, Baltimore, MD, United States

**Keywords:** human-animal interaction, healthy aging, functional status, BLSA, psychological status, wellbeing, quality of life, pet ownership

## Abstract

**Introduction:** Diminishing cognitive and physical functions, worsening psychological symptoms, and increased mortality risk and morbidity typically accompany aging. The aging population's health needs will continue to increase as the proportion of the population aged > 50 years increases. Pet ownership (PO) has been linked to better health outcomes in older adults, particularly those with chronic conditions. Much of the evidence is weak. Little is known about PO patterns as people age or the contribution of PO to successful aging in community-dwelling older adults. This study examines PO patterns among healthy community-dwelling older adults and the relationship of PO to cognitive and physical functions and psychological status.

**Methods:** Participants in the Baltimore Longitudinal Study of Aging (> 50 years old, *N* = 378) completed a battery of cognitive, physical function, and psychological tests, as well as a PO questionnaire. Descriptive and non-parametric or general/generalized linear model analyses were conducted for separate outcomes.

**Results:** Most participants (82%) had kept pets and 24% have pets: 14% dogs, 12% cats, 3% other pets. The most frequent reasons for having pets included enjoyment (80%) and companionship (66%). Most owners had kept the pet they had the longest for over 10 years (70%). PO was lower in older decades (*p* < 0.001). Pet owners were more likely to live in single-family homes and reside with others (*p* = 0.001) than non-owners. Controlling for age, PO was associated independently with better cognitive function (verbal leaning/memory *p* = 0.041), dog ownership predicted better physical function (daily energy expenditure, *p* = 0.018), and cat ownership predicted better cognitive functioning (verbal learning/memory, *p* = 0.035). Many older adults who did not own pets (37%) had regular contact with pets, which was also related to health outcomes.

**Conclusion:** PO is lower at older ages, which mirrors the general pattern of poorer cognitive and physical function, and psychological status at older ages. PO and regular contact with pets (including PO) are associated with better cognitive status compared with those who did not own pets or had no regular contact with pets independent of age. Dog ownership was related to better physical function. Longitudinal analysis is required to evaluate the association of PO and/or regular contact with maintenance of health status over time.

## Introduction

Poorer cognitive (1) and physical function (2), greater psychological symptoms (3, 4), and increased mortality and morbidity (5) typically accompany aging. With increasing numbers of adults aged 50 years or older over the next several decades, health needs in these areas will continue to increase. Successful aging includes living without disease and disability for as long as possible, while retaining cognitive and physical function, and psychological adaptation (6). The goal of successful aging, also known as healthy aging, is to live with the best function possible for as long as possible, hopefully reducing the current 5–7 year gap between high life quality and total life expectancy (7).

Interaction with animals is a non-pharmacological intervention that is posited to support health and may promote healthy aging (8, 9). Considerable research addresses the relationship between human-animal interaction and people's health (9, 10). Mechanisms for the benefits from human-animal interaction can be understood from the framework of the biopsychosocial model. In this model, biological, psychological, and social realms interact with each other to determine health outcomes. Health outcomes are conceptualized on a continuum and are influenced by negative (challenges) and positive (enhancements) alterations within each of the realms. Human-animal interaction can be conceptualized as an enhancement in the social realm, that in turn can improve psychological status through social support or other mechanisms, such as lower depression, stress, and anxiety, and may ultimately foster positive health outcomes.

Evidence suggests that two forms of human-animal interaction, pet ownership, and animal-assisted interactions (AAI), promote each of these aspects of health at some point in the human lifespan (10). A recent meta-analysis documented that the strongest evidence of benefits of HAI for older adults is from studies of AAIs (8). Animal-assisted therapy or the less structured animal-assisted activities may prevent or reduce depression, loneliness, and anxiety and optimize psychological health as well as encourage physical activity and promote physical and cognitive function (10–14).

The impact of pet ownership or regular interaction with others' pets on the health of older adults is less clear (8). Little is known about patterns of pet ownership or regular contact with others' pets in older adults. Pet ownership is common in community dwelling older adults with estimates ranging upward from 50% among individuals over the age of 50 (15). Evidence supports the contribution of pet ownership to some aspects of successful aging.

### Pet Ownership and Cognitive/Physical Function

Research links pet ownership with better health outcomes in older adults, particularly those with chronic health conditions that are common in older adults (8). The strongest evidence comes from studies examining cardiac health (8), where older adults with hypertension had lower blood pressure in the presence of their pets (16). In an analysis of 460 older adults who had experienced a myocardial infarction, pet ownership predicted better survival (17). The American Heart Association conducted a review of the existing evidence, carefully weighing all the results, and issued a statement that pet ownership, particularly dog ownership, probably plays a causal role in reducing the risk of cardiovascular disease (18). The evidence-base is not consistently positive about the link between pet ownership and cardiac health in older adults as one study examining hospital patients admitted for acute cardiac symptoms, found pet ownership, specifically cat ownership, related to higher mortality, or hospital readmission (19).

Another area that has received considerable attention is the potential benefit of exercising dogs on people's health. Obtaining a dog was related to increased walking (20). Several studies (21–23) suggest that walking dogs supports higher engagement in moderate physical activity, which may be related to physical function, a successful aging outcome. In one such study, compared to their non–dog owning matched counterparts, community-dwelling older adult dog-owners spent more time walking every day (average of 22 min), took 2,760 more steps per day, undertook their walking at a cadence necessary to achieve recommended levels of activity per day, and experienced fewer prolonged sedentary events each day (21). A meta-analysis indicates that dog-owners walk their dogs a median of four times per week totaling a median of 160 min (24). These findings support a direct link between dog walking and better physical function or fitness.

### Pet Ownership and Psychological Adaptation

Investigations of pet ownership and depression, anxiety, loneliness and social functioning, among others tend to link pet ownership with positive outcomes, but findings have been mixed, and most of the evidence is weak (8) with publication bias also a potential issue (25).

One theme running through studies of pet ownership and health concerns the possibility of systematic differences between people who own pets and those who do not. These differences may explain apparent differences in health outcomes, rather than pet ownership itself. Several approaches can address this issue; one is to identify and control for variables related to pet ownership in the statistical analysis, but this requires us to understand more about older adults' reasons for having and not having pets. It is also important to include regular pet contact in addition to pet ownership.

### Regular Contact With Pets

Studies support pet ownership as having a positive impact on the health of aging adults, but ownership may not be necessary for aging adults to benefit from human animal interaction (8). In fact, the evidence base for the positive impact of AAI on physical health, depression, anxiety, and loneliness for older adults is stronger than pet ownership research on the same outcomes. Few studies report the frequency older adults have regular contact with others' pets, and little is known about such contact in healthy older adults. This is particularly important because older adults face a number of challenges related to maintaining their pets (26).

### Attitudes Toward Pets

Researchers also hypothesize that the health effects that pets have on their owners relates to owner's attitudes toward their pets. One study of anti-arousal effects of dogs in college students found lower arousal during a stressor with a friendly dog present in students with positive attitudes toward dogs than those with less positive attitudes toward dogs (27). Krause-Parello (28) found attachment to pets was more important for those who expressed loneliness than those who reported adequate social support and further that the strength of attachment of older women to their pets mediated the relationship between loneliness and general health in community living older women.

### Reasons for Owning and Not Owning Pets

Older adults experience several challenges related to maintaining their pets as they age (26) and as a result many do not own pets, but little research delineates aversion to pet ownership. To best understand the potential of pet ownership for health benefits, it is necessary to appreciate existing barriers to pet ownership and why people do not own pets.

The current study was designed to learn more about (1) pet ownership and pet contact patterns in healthy community dwelling older adults and (2) the association of pet ownership with successful aging health outcomes.

The first aim of the current study was to learn more about pet ownership and human-animal interaction patterns among healthy community dwelling older adults and the factors that predict pet ownership among healthy older adults. We examined how pet ownership patterns varied as people aged and whether the patterns differed for cat and dog owners, for men and women, and for those who in different living situations (alone/with others and in own homes/apartments, etc.). Among pet owners, we explored whether reasons for owning pets, attachment to pets and influences of pets on people's lives differed according to participants' sex and species of their pet. We also explored differences in dog walking according to age and sex. Among those who do not own pets, we examined reasons for not owning a pet and the frequency of contact of with other people's pets.

The second aim of this study was to examine the association of pet ownership or regular contact with others' pets to health outcomes related to successful aging in healthy community dwelling older adults. We hypothesized that pet ownership would be associated with healthy aging outcomes after controlling for differences between pet owners and non-owners, and that dog walking would be associated with better physical function. The healthy aging related outcomes assessed were: (1) disability/disease (physical well-being); (2) maintaining cognitive (verbal learning and memory, visual perceptual motor speed) and physical function (gait speed, daily physical activity); and (3) psychological adaptation (psychological well-being, depression, anxiety, and happiness).

## Methods

### Design

The study used a cohort design with prospective health data obtained in the Baltimore Longitudinal Study of Aging (BLSA), an ongoing National Institute on Aging (NIA) Intramural Research Program funded cohort study. The BLSA is America's longest-running scientific study of human aging. Started in 1958, BLSA is a longitudinal observational study that addresses critical questions about normal and pathological age-related change. Researchers measure cognitive and physical changes associated with aging in real time during 3 consecutive days of testing at regular intervals over the course of participants' lives. Participants who are 20–60 years old complete the assessment every 4 years; those 60–79 years old complete it every 2 years and those 80 years and older complete it annually. Following IRB approval by the National Institute of Environmental Health Sciences (National Institutes of Health) Office of Research Compliance, Institutional Review Board, a group of pet ownership related questions were added to the battery of surveys completed during visits starting in March 2017. Data from participants aged 50 years and above at the time of assessment are included in this analysis.

### Participants

Over a 1-year period, 378 BLSA participants aged 50–101 completed the pet-ownership survey. As shown in Table 1, 56.9% were female, 59.5% married, 55.6% lived with one other person (55.6%), and 78.9% resided in a single-family home. Participants are highly educated with 64% having a postgraduate degree and non-poor with 70% reporting a family income over $50,000 per year.

**Table 1:** Demographic and pet ownership characteristics of respondents (*N* = 378).

**Characteristics**	**Category**	***N* (%)**
	**M(SD)**	**Range**
Visit Age (years)	76.9 (10.0)	50.8–100.8
Visit Number	8.5 (6.0)	1–31
Sex		
	Female	215 (56.9)
	Male	163 (43.1)
Race		
	Black	106 (28.0)
	White	252 (66.7)
Education		
	< High school	2 (0.5)
	High school grad	11 (2.9)
	Some college	38 (10.1)
	College grad	84 (22.3)
	Post grad degree	242 (64.2)
Income		
	< = $10K	6 (1.7)
	$11K to 25K	16 (4.6)
	$26K to 50K	61 (17.6)
	> $50K	263 (76.0)
Marital status		
	Married	213 (59.5)
	Living with Partner	1 (0.3)
	Separated	1 (0.3)
	Divorced	49 (13.7)
	Widowed	66 (18.4)
	Never Married	28 (7.8)
Number of others in household		
	None	116 (32.4)
	One Other	199 (55.6)
	Two Others	28 (7.8)
	> Three Others	15 (4.2)
Housing type		
	Single-family	280 (78.9)
	Co-Op, Condo, Apt	60 (16.9)
	Continuing care community	13 (3.7)
	Assisted living	2 (0.6)
Current pet owner		
	No	288 (76.2)
	Yes	90 (23.8)
Current dog owner		
	No	325 (86.2)
	Yes	52 (13.8)
How many dogs		
	1	36 (9.5)
	2	10 (2.6)
	3	2 (0.5)
	4	1 (0.3)
Current cat owner		
	No	330 (87.5)
	Yes	47 (12.5)
How many cats?		
	1	27 (7.1)
	2	14 (3.7)
	3	3 (0.8)
	4	1 (0.3)
Current small mammal owner		
	No	375 (99.7)
	Yes	1 (0.3)
Current bird owner		
	No	374 (99.5)
	Yes	2 (0.5)
Current fish owner		
	No	372 (99.0)
	Yes	6 (1.5)
Current reptile owner		
	No	376 (100.0)
	Yes	0 (0.0)
Current other pet owner		
	No	373 (99.2)
	Yes	3 (0.8)
Owned a pet in past 10 years		
	No	236 (62.4)
	Yes	142 (37.6)
Owned a dog in past 10 years		
	No	287 (75.9)
	Yes	91 (24.1)
Owned a cat in past 10 years		
	No	295 (78.0)
	Yes	83 (22.0)

Pet ownership related variables were assessed using multiple sources: (1) a 10-year pet-ownership history questionnaire designed for this study, (2) the pet ownership and interaction module from the NIA funded Health and Retirement Study (HRS) (29, 30), (3) the Lexington Attachment to Pets Scale (LAPS), and (4) questions about owners' perceptions of the influence pets have on their lives. The HRS pet-ownership module ascertained reasons for owning and not owning pets. Respondents could endorse as many options as they wanted from the 10-item list. Their most important reason for having or not having a pet was also queried. Individuals who owned dogs were also asked about dog walking behavior including whether they walked their dog, how frequently, and the duration of their walks. Participants who walked their dogs were asked to indicate how distance walked and speed walked with the dog related to their walking without the dog. In addition, the HRS module contains questions about regularity of contact with others' pets, type of pets, and walking behavior with others' pets.

The LAPS includes 23 items about individuals' attitudes toward their pets. Participants were asked to rate the degree to which they endorse each item on a 4-point Likert scale, choosing among strongly disagree, somewhat disagree, agree somewhat, or agree strongly. Two items are worded negatively and reversed for scoring. Items are then summed and averaged, with higher scores indicating greater attachment. The LAPS was validated in a representative population sample (α = 0.93) and was found strongly related to other indicators of pet attachment including personal reports and not related to social support from people (31). LAPS coefficient alpha in the current study is 0.84.

Pet owners were also asked about potential negative and positive effects of pet ownership suggested in previous reports and brainstorming discussions. Each question was scored on a 5-point Likert scale ranging from never to often and addressed the frequency with which the participant: (1) declined to visit family or friends or take a trip out of concern for their pet's welfare; (2) delayed or refused medical care out of concern for their pet's welfare while they were being treated; (3) experienced expenses related to their pet(s) that impacted their ability to pay for necessities for themselves and their families; (4) needed medical attention because they were scratched, bitten, or tripped by their pet(s); (5) found that having a pet(s) encourages them to be more socially active; and (6) found that having a pet(s) encourages them to take better care of their health. From these responses, we created a scale of the magnitude of the influence derived from factor analysis. A one factor solution was chosen based on the criteria of Eigen values of 1 or above. The scale was internally consistent (α = 0.70) and heterogeneous. Higher scores indicate owners perceiving that their pets have greater strength of influence on their lives.

Three categories of healthy aging-related outcomes were assessed: (1) lower disability/disease; (2) higher cognitive and physical function; and (3) higher psychological adaptation.

1) Disability/Disease

Lower disability/disease was conceptualized as higher health-related quality of life and assessed with the physical health subscale (PCS) of the Medical Outcome Study Short Form-12 (SF-12) (32). The SF-12 includes 4 yes/no questions and eight questions on Likert scales (up to six options). According to Center for Health Service Development (33) a meta-analysis instrument review revealed SF-12 to be a psychometrically sound tool with test-retest reliability for the PCS of 0.89. Higher scores on the PCS indicate better function and less disability (33).

2) Cognitive and Physical Function

Two dimensions of cognitive function were assessed. Verbal learning and memory were evaluated using the California Verbal Learning Test (CVLT) immediate (total) verbal recall defined as the total number of items recalled (out of 80 possible) across five learning trials. Visual perceptual motor speed was ascertained using the Digit Symbol Substitution Test (DSST) a component of the Weschler Adult Intelligence Scale-Revised (34). Participants were presented with a series of nine-digit symbol pairs and a string of digits and paired as many as possible within 90 s. Higher scores indicate better cognitive functioning (35). Data indicate good (0.71−*0.8*1) test-retest reliability (36). In the BLSA, neurocognitive function testing is performed by trained, certified examiners.

Measures of physical function include the rapid gait speed and the daily activity level. Rapid gait speed was assessed over a 6 m course with participants walking at their usual walking speed for 6 m twice and then at their maximum walking speed for 6 m twice. Time for each walk was measured with a stopwatch to the hundredth of a second. The time for the fastest of the two maximum speed walks was divided into 6 and provided the rapid gait speed (m/s). This test is validated as a measure of physical fitness by a negative correlation (−0.79) with peak VO_2_ (37, 38). The number of calories of activity in a day was based on physical activity estimates. Individuals were asked how many days per week they do varying intensity of physical activity and then how many minutes they do that type of activity per day. The estimated calories expended in each category were summed to provide estimates of daily energy expenditure (39).

3) Psychological Adaptation

Psychological adaptation was assessed by psychological well-being, depression, anxiety, and happiness. Psychological well-being was assessed with the mental health subscale (MCS) of the SF-12. The MCS, a score derived from the SF-12 is a well-validated measure of psychological well-being. Higher scores on the MCS indicate better psychological adaptation (40).

Depression was assessed with the Center for Epidemiologic Studies Depression Scale (CES-D). The CES-D consists of 20 items coded and scored according to scoring recommendations (41). It uses a four-point Likert scale, ranging from “rarely or none of the time” (0 point) to “most or all of the times” (three points) with four items reversed scored. Possible scores range from 0 to 60, with higher scores indicating a greater likelihood of depressive symptomatology (42–44). Validity in a nationally representative sample of non-institutionalized adults was established with internal consistency of 0.90 (45).

Anxiety was assessed with a subset of six items from the Perceived Stress Scale (PSS), a 10-item scale that measures the degrees to which life situations are appraised as stressful. A higher score on the PSS indicates higher anxiety. Happiness was derived from a single item with a range of 0–10. The higher the number, the greater the happiness. Good psychometric quality has been reported (46), and this scale was reliable (α = 0.83) in a community sample of older adults (47).

### Statistical Analysis

To analyse the first aim, bivariate categorical data were tested using Chi-square or Fisher's exact test when tables included expected cell sizes <5. Bivariate analysis of normally distributed variables was conducted using the Student's *t*-test or ANOVA. The Wilcoxon rank sum was used to test skewed continuous variables. Predictors of PO were examined using logistic regression. These non-directional questions were addressed with two-tailed analyses.

For the second aim, linear regression analyses were used to examine the contributions of pet ownership of various types to each health outcome. If ownership differed by age, and age predicted the outcome, age was controlled for and the interaction of age with pet ownership was included in an additional model to evaluate whether age modified the contribution of pet ownership to the successful aging outcome. The potential roles of dog walking and pet attachment as contributors to healthy aging outcomes were considered as a secondary aim. One-tailed analyses were conducted for the directional hypotheses of associations of pet ownership and dog walking with health outcomes. For the final aim, correlations were used to examine the relationships between attachment scores and health outcomes. These analyses were conducted with two-tailed analyses. All analyses were conducted with SPSS 25 (IBM Inc., Armonk, NY) or SAS (Carey, NC).

## Results

### Pet Ownership Patterns Among Older Adults

Most participants (81.7%) kept a pet at some time in their lives; 66.1% had a dog, 43.7% had a cat, 19.8% had fish, 13.0% had a small mammal, 10.3% had a bird, 6.9% had a reptile, and 3.7% had another pet. Many participants (37.6%) kept pets at some time during the past 10 years; 24.1% kept a dog, and 22.0% kept a cat. At the time of their BLSA visits, 23.8% currently had pets; 13.8% had dogs, 12.5% had cats, and 3.2% had other pets. Most pet owners kept the pet they had the longest for 10 or more years (70%).

### Demographic Variables Related to Pet Ownership

#### Age

As hypothesized, pet owners were significantly younger (*M* = 71.8, *SD* = 9.8 years) than non-owners [*M* = 78.54, *SD* = 9.44 years, *t*_(376)_ = 5.865, *t*_(376)_ = 5.87, *p* < 0.001]; and those who owned pets within the last decade were significantly younger (*M* = 73.6, *SD* = 9.9 years) than those who had not [*M* = 78.9, *SD* = 9.4 years, *t*_(376)_ = 5.236, *p* < 0.001]. The odds of owning a pet were lower by ~50% with each decade and the odds of owning a pet within the last 10 years were lower by about 40% with each decade (See Table 2).

**Table 2:** Percent of respondents who own any pet, a dog or a cat in each decade of age (years).

**Decade ownership**	**50s**	**60s**	**70s**	**80s**	**90s**	**OR10**	**95% CI**		***p***
PO C	50.0	35.3	28.2	14.4	0.0	0.52	0.41	0.67	*<0.0*01
PO 10	61.5	49.2	43.6	26.0	20.8	0.61	0.49	0.75	*<0.0*01
DO C	30.8	27.7	14.5	6.2	0.0	0.48	0.34	0.64	*<0.0*01
DO 10	50.0	36.9	26.5	13.7	12.5	0.56	0.44	0.07	*<0.0*01
CO C	23.1	20.0	13.7	8.2	0.0	0.61	0.45	0.82	*<0.0*01
CO 10	30.8	30.8	25.6	15.1	12.5	0.70	0.55	0.88	0.003

*OR10, odds ratio for having a pet 10 years later compared with having a pet at the referenced age decade; PO, pet owner; DO, dog owner; CO, cat owner; C, current; 10, within past 10 years*.

**Figure 1:**
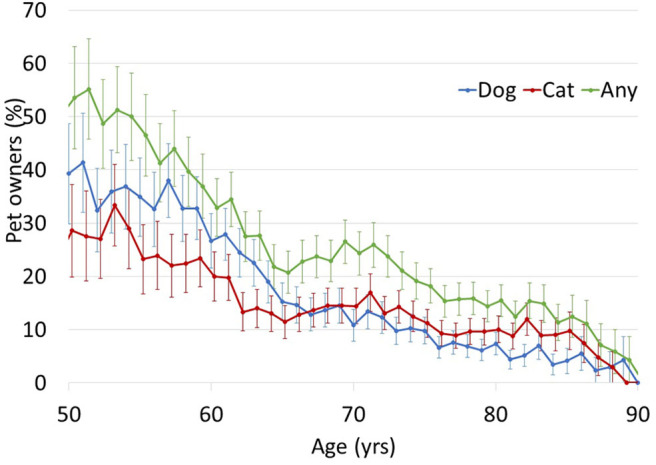
Frequency of owning any pet, a dog, and a cat in each age decade.

Dog and cat ownership were both lower with increasing age (see Figure 1). Current dog owners were significantly younger (age *M* = 69.6, *SD* = 9.4) than non-owners [age *M* = 78.0, *SD* = 9.5; *t*_(375)_ = 5.92, *p* < 0.001], and those who had owned a dog in the past 10 years also were significantly younger (age *M* = 71.6, *SD* = 10.1) than those who had not [age *M* = 78.6, *SD* = 9.3, *t*_(376)_ = 6.10, *p* < 0.001]. Current cat owners were significantly younger (age *M* = 72.5, *SD* = 10.0) than non-owners [age *M* = 77.5, *SD* = 9.8, *t*_(375)_ = 3.31, *p* = 0.001] as were those who owned cats in the past 10 years (age *M* = 74.2, *SD* = 9.8) compared with those who had not [age *M* = 77.7, *SD* = 9.9, *t*_(376)_ = 2.89, *p* = 0.004]. No one reported owning a dog or a cat in their 90s. The odds of owning a dog or a cat within the last 10 years was lower with each decade (Table 2). The odds of owning a cat became lower each decade (OR = 0.70) more slowly than the odds of owning a dog [OR = 0.49, *t*_(754)_ = −2.04, *p* = 0.021].

#### Sex

At the time of the assessment 26.5% of women and 20.3% of men owned pets, 15.0% of women and 12.3% of men had dogs, and 13.6% of women and 11.0% of men had cats. Within the last 10 years 39.5% of women and 35.0% of men owned pets, 24.6% of women and 23.3% of men owned dogs, and 23.7% of women and 19.6% of men owned cats. None of the pet ownership metrics (pet, dog, cat current ownership or pet, dog, cat past 10 years) differed by participant sex (see Table 3). Sex also did not moderate the differences in pet ownership metrics over time.

**Table 3:** Summary of regression analyses to examine the independent contributions of age, sex, and age*sex to pet ownership within the past 10 years and to current pet ownership.

**Pet**	**Model**	**Model effects**	**Age *p***	**Per decade OR**	**(95% CI)**		**Sex *p***	Age*sex *p*
**Pet ownership within the past 10 years**								
Any	1	Age	**<0.0001**	0.61	(0.49	to 0.75)	n/a	n/a
	2	Age, sex	**<0.0001**	0.61	(0.49	to 0.76)	0.532	n/a
	3	Age, sex, age*sex	**0.0008**	0.62	(0.47	to 0.82)	0.925	0.855
Dog	4	Age	**<0.0001**	0.56	(0.44	to 0.70)	n/a	n/a
	5	Age, sex	**<0.0001**	0.56	(0.44	to 0.70)	0.980	n/a
	6	Age, sex, age*sex	**0.0012**	0.60	(0.44	to 0.82)	0.463	0.461
Cat	7	Age	**0.003**	0.70	(0.55	to 0.88)	n/a	n/a
	8	Age, sex	**0.003**	0.70	(0.55	to 0.90)	0.441	n/a
	9	Age, sex, age*sex	**0.012**	0.68	(0.50	to 0.92)	0.616	0.694
**Current pet ownership**								
Any	1	Age	**<0.0001**	0.52	(0.41	to 0.67)	n/a	n/a
	2	Age, sex	**<0.0001**	0.53	(0.41	to 0.67)	0.268	n/a
	3	Age, sex, age*sex	**<0.0001**	0.51	(0.37	to 0.70)	0.618	0.734
Dog	4	Age	**<0.0001**	0.48	(0.36	to 0.64)	n/a	n/a
	5	Age, sex	**<0.0001**	0.48	(0.36	to 0.64)	0.668	n/a
	6	Age, sex, age*sex	**0.0003**	0.49	(0.34	to 0.72)	0.900	0.846
Cat	7	Age	**0.0008**	0.61	(0.45	to 0.81)	n/a	n/a
	8	Age, sex	**0.0001**	0.61	(0.45	to 0.82)	0.606	n/a
	9	Age, sex, age*sex	**0.003**	0.55	(0.37	to 0.81)	0.394	0.432

*p's are 2-tailed, bold indicates p <0.05*.

#### Housing Type

Pet ownership was significantly more frequent [Chi-square (df = 1) = 15.6, *p* < 0.001] among those who resided in single-family homes (28.6%) than in other types of housing (6.7%), as was pet ownership within the past 10 years [Chi-square (df = 1) = 14.7, *p* < 0.001]. Both dog and cat ownership were [Chi-square (df = 1) =8.0, *p* = 0.005; Chi-square (df = 1) =8.3, *p* =0.004, respectively] more frequent among residents of single-family homes (16.8, 15.0%) than of other housing (4.0, 2.7%). Dog ownership within the last 10 years did not differ significantly [Chi-square (df =1) = 3.7, *p* = 0.054] among residents of single-family homes (26.8%) than of other housing (16.0%); and cat ownership within the past 10 years was significantly more frequent [Chi-square (df = 1) = 6.2, *p* = 0.01] among residents of single-family homes (23.9%) than of other housing (10.7%).

#### Live Alone

Pet ownership was significantly lower [Chi-square (df = 1) =10.9, *p* = 0.001] among those who lived alone (12.9%) than those who resided with others (28.6%). Pet ownership within the past 10 years also was significantly lower [Chi-square (df = 1) =12.9, *p* < 0.001] among those who lived alone (24.1%) than those who lived with others (43.5%) at the time of the survey. Current dog ownership [Chi square (df = 1) = 8.8, *p* = 0.004] and cat ownership [Chi-square (df = 1) = 6.4, *p* = 0.012] were both significantly lower among those who lived alone than those who resided with others (dog ownership: 6%, 17.2%; cat ownership: 6%, 15.3%) as were dog ownership [Chi square (df = 1) = 8.1, p = 0.004] and cat ownership [Chi-square (df = 1) = 9.6, *p* = 0.002] within the past 10 years (dog ownership: 14.7%, 28.2%; cat ownership: 12.1%, 26.3%).

### Reasons for Pet Ownership

The most frequent reasons endorsed for currently having pets included enjoyment (83.3%) and companionship (65.6%). The reasons of enjoyment and companionship did not differ between participants whose favorite pet was a dog (enjoyment: 87.5%, companionship: 75%) vs. a cat (enjoyment: 80.0%, *p* = 0.37; companionship: 56.67%, *p* = 0.09) or by sex (women—enjoyment: 87.7%; women—companionship: 70.2%; men—enjoyment: 75.8%; *p* = 0.14; men—companionship 57.6%; *p* = 0.22). Keeping owners active or protected each were endorsed by ~20% of pet owning participants; with other reasons indicated by smaller proportions.

### Attachment to Pets

Scores on the LAPS ranged from 1.2 to 4.6 with a mean of 2.8 (SD = 0.66) and median of 2.8. There were no significant differences in average attachment [*t*_(75)_ = 1.2, *p* = 0.24] or variability of attachment [*F*_(28, 47)_ = 1.38, *p* = 0.32] between those with dogs (Mean = 2.9, SD = 0.6) and those with cats (Mean = 2.7, SD = 0.7) as their favorite pets. Attachment scores were not significantly different [*t*_(84)_ = 1.94, *p* = 0.055] nor did they differ in variability [F_(53, 31)_ = 1.9, *p* = 0.056] between women (Mean =2.9, SD = 0.7) and men (Mean = 2.7, SD = 0.5). Pet attachment was not related to age (*r* = −0.04, *p* = 0.77) or to any measure of successful aging (Table 4). Pet attachment did not differ [*t*_(84)_ = 1.6, *p* = 0.11] between participants who lived alone (Mean = 3.1, SD = 0.77) and those who lived with others (Mean = 2.8, SD = 0.6).

**Table 4:** Correlation of pet attachment with measures of successful aging among current pet owners.

**Variable (N)**	***r***	***p***
**Disease/disability**		
Physical well-being (98)	−0.02	0.915
**Cognitive function**		
Verbal learning/memory (106)	0.06	0.576
Visual perception (104)	0.005	0.963
**Physical function**		
Rapid gait speed (106)	−0.05	0.628
Daily energy expended (103)	0.08	0.430
**Psychological adaptation**		
Psychological well-being (98)	−0.05	0.648
Depression (106)	0.03	0.726
Anxiety (109)	0.10	0.287
Happiness (104)	−0.00	0.970

*p's are 2 tailed*.

Among current pet owners, a single item question asking if their pets made them happy was agreed to strongly in 48.1% and agreed to somewhat in 39.6% of current pet owners. Similarly, among past or present owners, 85.0% indicated that pets contributed to their happiness. Significantly more [Chi-square (df = 1) = 4.9, *p* = 0.032] older individuals (90.4% above median age) than younger individuals (80.7%) reported that their pets contributed to their happiness.

### Influence of Pets on Owners' Lives

Most people indicated that negative effects of pet ownership occurred infrequently (never or almost never) and positive influences occurred frequently (see Table 5), Dogs were more likely (36.0%) than cats (12.0%) both to facilitate social interaction (Wilcoxon rank-sum test *p* = 0.01) and to cause owners to decline visits to family members (dogs: 30% cats: 21.0%; Wilcoxon rank-sum test *p* = 0.04).

**Table 5:** Pet owners' perceptions of how their pets influence their lives.

	**Any pet owners**		**Dog owners**		**Cat owners**	
	**Rarely^†^ (*n*) %**	**Some (*n*) %**	**Rarely (*n*) %**	**Some (*n*) %**	**Rarely (*n*) %**	**Some (*n*) %**
Declined to visit family or friends or take a trip out of concern for your pet's welfare	(87) 76.3	(27) 23.7	(45) 70.3	(19) 29.7	(27) 79.4^*^	(7) 20.6^*^
Delayed or refused medical care out of concern for your pet's welfare while you were being treated	(113) 99.1	(1) 0.9	(64) 100	(0) 0	(34) 100	(0) 0
Expenses related to your pet(s) impacted your ability to pay for necessities for yourself and your family	(109) 96.5	(4) 3.5	(63) 98.4	(1) 1.6	(30) 90.9	(3) 9.1
Needed medical attention because you were scratched, bitten, or tripped by your pet(s)	(110) 97.4	(3) 2.7	(62) 98.4	(1) 1.6	(32) 94.1	(2) 5.9
	**Little^‡^ (*n*) %**	**More (*n*) %**	**Little (*n*) %**	**More (*n*) %**	**Little (*n*) %**	**More (*n*) %**
Having a pet(s) encourages you to be more socially active	(83) 73.4	(30)26.6	(41) 64.1	(23) 35.9	(29) 87.9^*^	(4) 12.1^*^
Having a pet(s) encourages you to take better care of your health	(88) 79.3	(23) 20.7	(48) 75.0	(16) 25.0	(26) 81.3	(6) 18.8

† *Rarely, never or almost never; Some, a few times, several times, or often*.

‡ *Little, not at all or a little; More, some or a lot*.

* *p < 0.05, difference between owners and non-owners, p's are 2-tailed*.

Scores on the combined scale of the magnitude of the influence of pets on their owners' lives ranged from −0.7 to 3.1 with a mean of 0.0 and SD of 0.9. The combined influence scale was not correlated with age [*r*_(111)_ = −0.002, *p* = 0.99] indicating that the influence may not change over time. Scores of women (*N* = 51, *M* = 0.05, SD = 0.96) and men (*n* = 31, *M* = −0.03, SD = 0.81) did not differ [*t*_(80)_ = 0.39, *p* = 0.70]. Pet owners with a dog as their favorite pet (*n* = 13) did not indicate that their favorite pet [*t*_(36)_ = 2.0, *p* = 0.055] had more influence on their lives (*M* = 0.5, SD = 1.0) than pet owners with a cat as their favorite pet (*n* = 25, *M* = −0.1, SD = 1.0). Pet attachment was significantly related to the influence owners perceived their pets have on their lives [*r*_(100)_ = 0.46, *p* < 0.001]; higher attachment was related to greater influence on owners' lives.

### Dog Walking

Of the 52 current dog owners, 37 (71.2%) walk their dogs. Dog walking did not differ by sex (women: 23/29, 79.31%; men: 14/20, 70.00%; Fisher's exact *p* = 0.51). Dog walking also was not related to age [50–59 years: 5/7 (71.4%); 60–69 years: 15/18 (83.3%); 70–79 years: 70–79: 12/16 (75.0%); 80–89 years: 5/8 (62.5%); 90–99 years: 0/0; Fisher's exact *p* = 0.67].

A majority of dog owners (60%) indicated they walked more because they owned dogs; 26% walked a lot more and 34% walked somewhat more. In contrast, 34% walked about the same amount and 6% walked less because they owned dogs. Dog owners who walked their dogs generally reported walking about the same speed (26.5%) or slower (55%) than when they walked without the dog. Those who walked their dog generally reported walking further with their dog (40.8%) or about the same distance (26.5%) with their dog compared with when they walked without their dog.

### Reasons for Not Having a Pet

**Figure 2:**
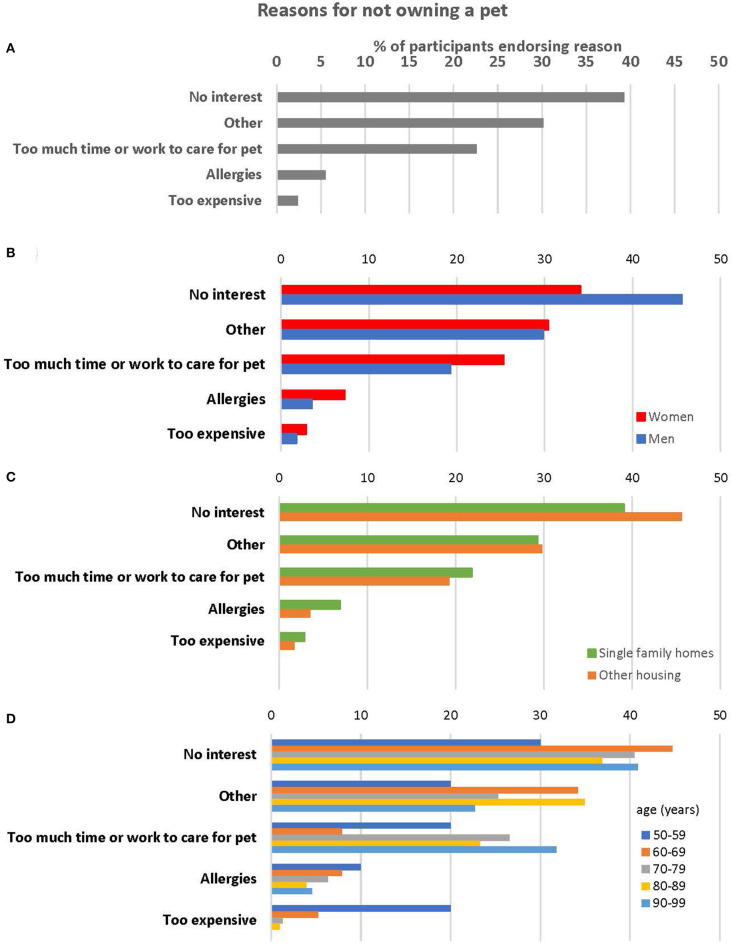
Reasons for not owning a pet for **(A)** All respondents who do not own a pet (*n* = 288); **(B)** According to respondent sex; **(C)** According to housing type; and **(D)** According to age decade.

A large majority (76%) of participants did not own pets at the time of their BLSA assessment. The most cited reasons for not having a pet was lack of interest in owning a pet (39%) and the time or work it takes to care for a pet (23%), with small numbers indicating their or family members' allergies (6%), or expense kept them from having a pet (2%; see Figure 2). The option “Other” was endorsed as a reason for not owning a pet by 76% of those who did not keep pets. Unfortunately, further details about what these other reasons might be are not available. Reasons men and women did not own pets did not differ [Chi-square (df = 4) = 5.1, *p* = 0.28]. Participants' reasons for not owning pets differed by age (*p* = 0.02), with those citing expense being significantly younger (67.3 years, 95% CI 58.9–74.6 years) than those citing other reasons. Those citing no interest had an estimated age of 78.6 years (95% CI 76.8–80.4 years), those citing pets require too much time or work to care for had an estimated age of 79.6 years (95% CI 77.2–82.0 years), those citing allergies had an estimated age of 75.0 (95% CI 70.2–79.9 years) and those citing “Other” had an estimated age of 78.8 years (95% CI 76.8–80.9 years). Participants who lived in single-family homes did not differ from those in other types of housing in the reasons they did not own pets [Chi-square (df = 4) = 3.45, *p* = 0.49]. Similarly, reasons did not differ for those who live alone and those who live with others (Fishers' *p* = 0.26).

### Regular Contact With Pets Among Non-owners

Approximately 37% (*n* = 106) of non-owners reported regular contact with others' pets. Non-owners' contact with other's pets did not vary by sex [Chi-square (df = 1) = 2.53, *p* = 0.11], with 48.2% of women and 38.0% of men having regular contact with other's pets. Non-owners' contact with other's pets did not vary by age [Chi-square (df = 4) = 7.21, *p* = 0.12], with 40% of those in their fifties, 29.7% of those in their sixties, 38.2% in their seventies, 52.4% in their eighties, and 47.1% in their nineties having contact with other's pets. Approximately 36% (*n* = 36) of those who had regular contact with a pet they did not own indicated that they walked with someone else's dog, and 6% (*n* = 6) walked a dog more than once per week.

### Association of Pet Ownership With Successful Aging Outcomes

Indicators of disease/disability, cognitive/physical function and psychological adaptation were examined in relation to current pet (yes: *n* = 90, no: *n* = 288), current dog (yes: *n* = 52, no: *n* = 325), and current cat ownership (yes: *n* = 47, no: *n* = 330), pet ownership within the past 10 years (yes: *n* = 142, no: *n* = 236), regular contact with a pet (yes: *n* = 223, no: *n* = 155), dog ownership within the past 10 years (yes: *n* = 91, no: *n* = 287), and dog walking among dog owners (yes: *n* = 37, no: *n* = 12). Findings are summarized in Tables 6, 7. Additional details of these analyses are included in Supplementary Tables 1–7.

**Table 6:** Summary of contribution of current pet ownership, dog ownership, and cat ownership to measures of successful aging in bivariate analysis and controlling for age.

**Predictor**		**PO**		**DO**		**CO**	
**Health outcome**	**Model**	**PO *p***	**Age *p***	**DO *p***	**Age *p***	**C0 *p***	**Age *p***
**Disease/disability**							
Physical wellness	PET	**0.026**	N/A	0.010	N/A	0.123	N/A
	PET, age	0.145	**0.018**	0.316	**0.011**	0.300	**0.009**
**Cognitive function**							
Verbal learning/memory	PET	**0.0002**	N/A	**0.0009**	N/A	**0.002**	N/A
	PET, age	**0.041**	**<0.0001**	0.077	**<0.0001**	0.052	**<0.0001**
Visual Perception	PET	**<0.0001**	N/A	**0.008**	N/A	**0.002**	N/A
	PET, age	0.076	**<0.0001**	0.450	**<0.0001**	0.052	**<0.0001**
**Physical function**							
Rapid gait speed	PET	**0.0002**	N/A	**0.0002**	N/A	**0.004**	N/A
	PET, age	0.139	**<0.0001**	0.097	**<0.0001**	0.160	**<0.0001**
Daily energy expenditure (Kcal)	PET	**0.0002**	N/A	**<0.0001**	N/A	**0.016**	N/A
	PET, age	0.054	**<0.0001**	**0.018**	**<0.0001**	0.305	**<0.0001**
**Psychological adaptation**							
Psychological Well-being	PET	0.198	N/A	0.401	N/A	0.235	N/A
	PET, age	0.400	0.107	0.239	0.071	0.361	0.098
Depression	PET	0.494	N/A	0.111	N/A	0.096	N/A
	PET, age	0.290	**0.023**	0.212	**0.036**	**0.042**	**0.015**
Anxiety	PET	0.099	N/A	0.449	N/A	0.082	N/A
	PET, age	0.277	0.106	0.229	0.052	0.174	0.097
Happiness	PET	0.261	N/A	0.130	N/A	0.340	N/A
	PET, age	0.212	**0.002**	0.113	**0.002**	0.344	**0.002**

*PET, the specific pet ownership variable as defined in the column heading; age, age decade; PO, pet ownership; DO, dog ownership; CO, cat ownership. Scales, Physical Well-being, Short Form-12 Physical Component Score (SF-12 PCS); Verbal learning/memory, California Verbal Learning Test total correct answers; Visual perception, Weschler Adult Intelligence Scale- Revised Digit Symbol Substitution Test total score; Psychological well-being, Short Form-12 Mental Component Score (SF-12 MCS); Depression, Center for Epidemiologic Studies Depression Scale (CES-D) score; Anxiety, Perceived Stress Scale total score; Happiness, single item (1–10). p's are 1-tailed, bold indicates p < 0.05*.

**Table 7:** Summary of contribution of pet ownership within last 10 years (10), regular contact with a pet (PC), dog ownership within the last 10 years (DO10), and dog walking (DW) to measures of successful aging in bivariate analysis and controlling for age.

**Predictor**		**PO10**		**PC**		**DO10**		**DW**	
**Health Outcome**	**Model**	**PO10 *p***	**Age *p***	**PC *p***	**Age *p***	**DO10 *p***	**Age *p***	**DW *p***	**Age *p***
**Disease/disability**									
Physical wellness	PET	**0.025**	N/A	0.074	N/A	0.139	N/A	0.400	N/A
	PET, age	0.125	**0.017**	0.190	**0.010**	0.387	**0.009**	0.327	0.121
**Cognitive function**									
Verbal learning/memory	PET	**0.0002**	N/A	**0.0003**	N/A	**0.005**	N/A	0.472	N/A
	PET, age	**0.035**	**<0.0001**	**0.006**	**<0.0001**	0.136	**<0.0001**	0.432	0.262
Visual perception	PET	**0.002**	N/A	**0.0005**	N/A	**0.020**	N/A	0.332	N/A
	PET, age	0.206	**<0.0001**	**0.006**	**<0.0001**	0.357	**<0.0001**	0.152	**0.008**
**Physical function**									
Rapid gait speed	PET	**0.0001**	N/A	**0.0035**	N/A	**<0.0001**	N/A	0.319	N/A
	PET, age	0.068	**<0.0001**	<0.0001	0.108	**0.035**	**<0.0001**	0.462	**0.002**
Daily energy expenditure (Kcal)	PET	**0.004**	N/A	0.148	N/A	**0.0003**	N/A	0.435	N/A
	PET, age	0.236	**<0.0001**	0.457	**<0.0001**	0.077	**<0.0001**	0.352	**0.0002**
**Psychological adaptation**									
Psychological well-being	PET	0.4032	N/A	0.070	N/A	0.340	N/A	0.334	N/A
	PET, age	0.376	0.083	0.119	0.112	0.155	0.060	0.354	0.406
Depression	PET	0.471	N/A	0.310	N/A	0.251	N/A	0.421	N/A
	PET, age	0.277	**0.022**	0.422	**0.028**	0.390	**0.030**	0.458	0.388
Anxiety	PET	0.056	N/A	0.065	N/A	0.197	N/A	0.336	N/A
	PET, age	0.171	0.119	0.120	0.091	0.427	0.084	0.270	0.178
Happiness	PET	0.177	N/A	0.088	N/A	0.067	N/A	0.122	N/A
	PET, age	0.142	**0.0018**	0.099	**0.002**	**0.0344**	**0.0012**	0.1005	0.382

*PET, the specific pet ownership variable as defined in the column heading; age,age decade; PO10, pet ownership within the last decade; PC, pet ownership within the past 10 years or regular contact with a pet; DO10, dog ownership within the past 10 years; DW, walks dog (for current dog owners only). Scales, Physical Well-being, Short Form-12 Physical Component Score (SF-12 PCS); Verbal learning/memory, California Verbal Learning Test total correct answers; Visual perception, Weschler Adult Intelligence Scale- Revised Digit Symbol Substitution Test total score; Psychological well-being, Short Form-12 Mental Component Score (SF-12 MCS); Depression, Center for Epidemiologic Studies Depression Scale (CES-D) score; Anxiety, Perceived Stress Scale total score; Happiness, single item (1-10). p's are 1-tailed, bold indicates p < 0.05*.

In bivariate analysis, current pet ownership was associated with physical wellness (*p* = 0.026), better cognitive function (verbal learning/memory, *p* = 0.0002), visual perception, *p* < 0.0001)] and better physical function (rapid gate speed, *p* = 0.0002, daily energy expenditure, *p* = 0.0002). After controlling for age, pet ownership did not make a significant contribution to any health outcomes except cognitive function (verbal learning/memory, *p* = 0.041). In bivariate analysis, dog and cat ownership also were associated with cognitive (dog: verbal learning/memory, *p* = 0.0009; visual perception, *p* = 0.008; cat: verbal learning/memory, *p* = 0.0002, visual perception, *p* = 0.002) and physical function (dog: rapid gate speed, *p* = 0.0002, daily energy expenditure, *p* < 0.0001; cat: rapid gate speed, *p* = 0.004, daily energy expenditure, *p* = 0.016). After controlling for age, pet ownership was associated independently with better cognitive function (verbal leaning/memory *p* = 0.041), and dog ownership was associated with better physical function (daily energy expenditure, *p* = 0.018*)*. Estimated means are available in Supplementary Tables 1–3.

In bivariate analysis, pet ownership within the past 10 years was associated with less disease/disability, but dog ownership within the past 10 years, regular contact with pets, and dog walking (among dog owners) was not. After controlling for age, none of these pet-related variables independently predicted disease/disability.

In bivariate analysis, pet ownership within past 10 years (verbal learning/memory, *p* = 0.0002; visual perception, *p* = 0.002), regular contact with pets (verbal learning/memory, *p* = 0.0003; visual perception, *p* = 0.0005), and dog ownership within the past 10 years (verbal learning/memory, *p* = 0.005; visual perception, *p* = 0.020) predicted better cognitive function, but dog walking (verbal learning/memory, *p* = 0.47; visual perception, *p* = 0.33) did not (Table 7, Supplementary Tables 4–7). After controlling for age, pet ownership within the past 10 years (verbal learning/memory, *p* = 0.035) and regular contact with a pet (verbal learning/memory, *p* = 0.006; visual perception, *p* = 0.006) were significant independent predictors of better cognitive function. None of the other pet-related variables independently predicted cognitive function.

In bivariate analysis, pet ownership within past 10 years (rapid gait speed, *p* = 0.0001; daily energy expenditure, *p* = 0.004), regular contact with pets (rapid gait speed, *p* = 0.004), and dog ownership within the past 10 years (rapid gait speed, *p* < *0.0*001; daily energy expenditure, *p* = 0.0003) predicted better physical function, but dog walking did not (rapid gait speed, *p* = 0.319; daily energy expenditure, *p* = 0.435) (Table 7, Supplementary Tables 4–7). After controlling for age, dog ownership within the past 10 years independently predicted physical function (rapid gait speed, *p* = 0.034).

In bivariate analysis, pet ownership within past 10 years, regular contact with pets, dog ownership within the past 10 years, and dog walking did not predict psychological adaptation (Table 7, Supplementary Tables 1–4). After controlling for age, dog owners were happier than non-owners (*p* = 0.034). Happiness and psychological well-being were moderately correlated (*r* = 0.36, *p* < 0.001).

Among current pet owners, a single question asking if their pets made them happy was agreed to strongly in 48.1% and agreed to somewhat in 39.6% of current pet owners. Similarly, among pet owners past or present, 85.0% of the respondents indicated that pets contributed to their happiness. Significantly more [Chi-square (df = 1) = 4.86, *p* = 0.032] older individuals (90.4% above median age) than younger individuals (80.7%) reported that their pets contributed to their happiness.

## Discussion

The findings from this study support prior research exploring pet ownership among older adults. Among this sample of community dwelling older adults, pet ownership was associated with younger age, living in single-family homes, and living with others. It did not differ according to sex.

Patterns were similar for dog ownership and cat ownership. There are many potential explanations for the decline in pet ownership across age, but it is consistent with reports elsewhere that older adults are frequently faced with challenges associated with keeping pets (26).

Frequency of dog ownership (13.8% US; 18.0% UK) was slightly lower than in the British Longitudinal Study of Aging (48) while frequency of cat ownership (12.8% US; 12% UK) was similar. The most frequent reasons given for pet ownership in this group of older adults were enjoyment and companionship. This finding is consistent with the finding that the most frequent reason for having a pet was avoidance of loneliness in two studies of younger and middle-aged adults (49, 50) and the association of dog companionship with decreased perceptions of loneliness in older adults (51). These results suggest that pet ownership is used as a means of social support across age groups. Previous studies indicated differences in reasons for having pets between men and women at earlier stages in their lives, with men using pets more to keep active and less to provide social support (50). No sex differences were observed in the current study, suggesting that older adult men may seek social support from companion animals as much as women do or have a greater need for social support than when they were younger. The second most common reason given for keeping a pet in the previous studies of college faculty, students, and community members was keeping their owners active. This response was chosen by over 20% of the respondents who were asked to choose one reason for having a pet. This reason was not frequent among the older adults in the current study, who could choose multiple reasons for keeping a pet, suggesting that older adult pet owners rely less on their pets to keep them active.

It is important to note that in these cross-sectional data, pet owners were in general younger than those who did not own pets. This emphasizes the need to adjust for age differences when examining the contribution of pet ownership to successful aging outcomes. It also suggests that barriers to pet ownership increase as people age.

Authors often speculate about reasons older adults do not own pets [e.g., (26, 52)]. Key reasons cited include expenses, fears of health risk, and housing limitations, especially for those who live in senior housing. In the current study, the most cited reason for not having a pet, given by about 40% of non-owners, was a lack of interest. Time and effort associated with pet ownership was given as an important reason (23%) for not owning pets. This suggests that older adult pet owners understand the demands of responsible pet ownership and are willing to limit their responsibilities. A small percentage (6%) cited allergies and only 2% indicated that health risks were a reason not to have a pet. The low number of individuals who cited expense as a reason may have been a result of the relative affluence of the current sample compared with the general population. Reasons not explored in the current study included lack of housing that will accommodate pets and concerns about what will happen to the pet if the owner becomes disabled or dies. Housing issues are commonly given reasons for relinquishing of pets, although less frequent than pet aggression (52). Housing issues are not likely an important reason for not having pets in this sample in that over 70% lived in single-family homes. We suggest that these reasons should be added as options to future explorations of barriers to pet ownership for older adults.

Pet ownership can affect individual's behaviors related to their safety and health (53, 54). For example, pet owners' failure to evacuate during storms because they couldn't take their pets with them led to changes in emergency preparedness plans (55). Anecdotal reports also indicate that pet owners may prioritize their pets' needs above their own. In the current study a few pet owners reported having to sacrifice taking care of their own medical needs due to concerns about their pets (0.9%) or not being able to pay for items for themselves due to paying for their pets' care (3.6%). This was an affluent sample, in generally good health, and often living with another person. These limitations may be more frequent among older adults who are less affluent, more impaired, and/or living alone. In addition, ~25% of respondents did not visit friends or family because of concern for their pet's welfare and 3.7% of pet owners had been injured more than “almost never” by their pets. These questions should be asked in a broader range of the older adult population. This information is necessary to inform development of alternative arrangements and policies to support pet owners during times of difficulties as well as their normal lives.

Most previous examinations of the relationship of pet ownership to health-related outcomes in community living older adults uses pet ownership data based on current pet ownership or lifetime (ever) pet ownership. While this study was cross-sectional in nature, the questions about 10-year pet ownership history allowed us to get a more expansive look at pet ownership across the aging process. Recognizing that an instantaneous look at pet ownership status is not the best way to evaluate the influence of pet ownership on health, we were able to examine whether pet ownership within the last 10 years was related to successful aging outcomes in our sample. This is a step toward a longitudinal approach to simultaneous examination of pet ownership and successful aging outcomes.

In this study, current pet ownership and pet ownership over the last 10 years were related to cognitive function but not to disease, disability, physical function, or psychological adaptation independent of age. The finding that cognitive function was better in pet owners and those with regular contact with pets was different than the lack of difference in the large British aging cohort study where no relationship was found (48). In that study, there also was no relationship of pet or dog ownership to physical function, a finding similar to the current study. The finding that pet ownership was not related to happiness or psychological well-being was similar to that in the British cohort study (48) and a smaller Australian study (56). In contrast in a younger population, Bao and Schreer (57) found that dog owning was associated with well-being. In the current study neither cat nor dog ownership was independently associated with psychological well-being. This difference in findings could be a result of differences in scales used to measure the constructs. In the current study, happiness was measured with one item and a range of 0–10, while the previous study used a 4-item scale. Different type of scales can lead to conflicting results. However, the similarity of findings from both the MCS and the happiness scale support the validity of these findings for this community-resident relatively healthy group of older adults.

In the current study, there was no relationship of pet ownership to psychological adaptation independent of age. This negative finding is important in understanding the difference between the relations of pet ownership to health outcomes in healthy older adults and the results of animal assisted interventions. Reductions in depression is the most consistent finding in studies of animal assisted interventions for older adults in care homes (8). A longitudinal study would help understand the timing. It is possible that older adults acquire cats, independent of age, because they are depressed. A longitudinal study would help understand the timing of the depression and pet acquisition.

When examining the relationship of pet ownership to health outcomes, definitions of pet ownership can be inconsistent or problematic (9, 58) Individuals often own several types of pets and the research questions may be addressing different aspects of pet ownership related to specific outcomes. For example, researchers may wish to examine the influence of pets on physical fitness related to walking, and therefore consider dog owners to be anyone who owns a dog but not a cat and cat owners as anyone who owns a cat but not a dog. In the current study pet ownership was defined in different ways, depending on the purpose of the analysis. In separate sets of analyses we compared current pet owners with people who did not own a pet at the time of assessment, people who had owned pets within the past 10 years with people who had, people who had owned dogs in the past 10 years with people who had, and individuals who either owned a pet or had regular contact with a pet with people who did not own or have regular contact with a pet. Since a number of individuals owned both cats and dogs at the time of initial assessment (*n* = 17) or owned cats and dogs (*n* = 38) within the past 10 years, in analyses of attachment to pets, individuals were asked to answer questions about the pet they identified as their favorite pet and to indicate the species of that pet.

The complex relationship between psychological adaptation and pet attachment was demonstrated in the current study. Regular contact with pets was related to happiness and to psychological wellness, after controlling for age, but not among current pet owners. This contrasts with the findings of lower life satisfaction overall in pet owners, but higher satisfaction of pet ownership with barriers to social participation (59). In the current study, comparable analyses were not meaningful as social support *per se* was not measured. When living alone was considered a proxy for social isolation, only 20 of the 116 individuals who lived alone owned pets. Pet attachment was negatively associated with both depression and well-being in the pet owners who lived alone, but only with depression in pet owners who lived with others.

The current study produced some evidence of the potential benefit of exercising dogs for people's health. Approximately 75% of dog owners indicated that they walked their dogs; and 60% of dog owners indicated that they walked more because they had a dog than they would have otherwise. In bivariate analysis, physical function was better among pet owners than non-owners, among those who had regular contact with pets than those who did not, and among dog owners than non-owners. It may indicate that individuals who choose to keep dogs are healthier, whether they walk their dogs or not. Individuals who currently had dogs expended more energy in the day than those who did not, independent of age. Individuals with regular contact with pets had greater rapid gait speed than those who did not, and rapid gait speed was faster among those who owned dogs in the past 10 years than those who had not, after controlling for age. Several previous studies that suggest that walking dogs is related to successful aging outcomes indicate dog owners had fewer prolonged sedentary events each day (21), and spent less time in sedentary activities (60). The differences in findings in the current study may be a reflection of different methods of measurement of activity as the BLSA used a questionnaire to estimate amount of physical activity while some of the data in the aforementioned studies used accelerometer data to obtain direct measurements of physical activity.

The information about dog walking in the study indicated that walking with a dog generally did not lead people to walk faster, in fact most walked more slowly. Walking with a dog also did not lead to owners walking for shorter distances than walking without the dogs. The implication is dog walkers are spending more time walking when walking their dogs. Spending more time could be consistent with activities related to dog walking and to social interaction. In a previous study, younger individuals have more social interaction when walking with their dogs than when walking alone (61, 62). Dog-walking may also play a role in the social facilitation older dog owners experienced while walking dogs (63).

## Limitations

The current study provides valuable insight into pet ownership patterns in older adults, it also includes limitations that must be considered when interpreting the results. First, the sample is not representative of the US population of older adults. The participants tended to be affluent, healthy, and living with someone else. Socio Economic Status (SES) has been identified as a confounder in previous research on pet ownership in the Avon Longitudinal Study of Parents and Children (64) but because the current sample did not include a wide range of SESs, we were unable to examine this potential confounder. Because the sample fails to adequately represent the full spectrum of each of these characteristics/variables, all of the affiliated correlations may be smaller than they would have been if the entire range of the variable had been included in the sample. We recognize this limitation and encourage future investigators to use population representative studies for similar explorations.

The questions we could ask about pet ownership were limited, due to practical limitations (e.g., survey burden). We included questions about pet ownership in general, with some detail around interactions with their pets (e.g., play with, talk to, feeding) and dog walking in a manner parallel to that used in the Health and Retirement Survey to allow potential joining of data for research purposes. Although, we did ask about who is responsible for pet care, we did not ask about the amount of care they gave to their pets.

As a result, there is more information we would have liked to acquire from our participants including detailed information on how and in what context older adults interact with their own pet and/or other companion animals. We included some items related to these interactions, but our results and conclusions are limited to the items we were able to include. Also related to limitations on item-inclusion, is the fact that the pet ownership questions are being phased into the BLSA via data collection waves. This means that the sample size will continue to grow with each wave of data collection, but it also means that the full set of BLSA participants have not yet answered the pet ownership questions.

The analyses conducted in this paper focus on pet ownership, dog ownership, and cat ownership. While we would like to examine the contributions of individual types of pets to successful aging outcomes, the low frequency of pets other than cats or dogs precluded that. We recognize this limitation but have appropriately powered our study for the pet ownership analyses reported herein. We recognize a lack of power for comparisons of dog owners who walk and do not walk their dogs.

## Conclusion

The findings from this study suggest that pet ownership patterns in older adults may be related to their trajectory of change in successful aging outcomes, particularly for cognitive functioning, but not for disease, disability, physical function, or psychological adaptation. Evidence indicates pet ownership declines with advancing age as do physical, cognitive, and psychological function. This study provides a description of pet ownership amongst older adults, indicating that they frequently opt to own pets to ameliorate loneliness and bolster social support, but not as a way of staying active. Participants who chose not to own a pet most frequently reported a lack of interest with their combined responses indicating that they understand the demands of responsible pet ownership and wish to limit such demands on their time. Pet ownership was associated with better health outcomes even after accounting for the contribution of lower age to the health outcomes. In this study dog walking was not associated with better disease/disability, cognitive/physical function, or psychological adaptation. Additional longitudinal analysis is required to evaluate the association of pet ownership with successful aging outcomes.

## Data Availability Statement

The datasets presented in this article are not readily available because the study is ongoing and data are the property of the National Institutes on Aging through an application process. Requests to access the datasets should be directed to https://www.blsa.nih.gov/how-apply.

## Ethics Statement

The studies involving human participants were reviewed and approved by National Institute of Environmental Health Sciences (National Institutes of Health) Office of Research Compliance Institutional Review Board. The patients/participants provided their written informed consent to participate in this study.

## Author Contributions

EF conceived the protocol and analysis and interpretation. ES and SS facilitated collection of the data. EF and EB analyzed the data. EF and NG wrote the manuscript. All authors participated in refinements of protocol and/or analyses, contributed to the revision of the manuscript, and approved the final version of the manuscript for submission.

## Conflict of Interest

At the time the project was funded, NG was employed by WALTHAM. Neither NG nor WALTHAM were involved in data collection or analysis. The remaining authors declare that the research was conducted in the absence of any commercial or financial relationships that could be construed as a potential conflict of interest.
