# Supplementary material for: Pet Ownership Patterns and Successful Aging Outcomes in Community Dwelling Older Adults
Source: Front Vet Sci. 2020 Jun 25;7:293. doi: 10.3389/fvets.2020.00293 (PMC7330097; doi:10.3389/fvets.2020.00293)
Supplement: Supplementary file 1 [file Data_Sheet_1.docx]

| **Supplementary Table 1**. Summary of Contribution of Current Ownership (PO) to Measures of Successful Aging in Bivariate Analysis and Controlling for Age | | | | | | | | |
| --- | --- | --- | --- | --- | --- | --- | --- | --- |
|  |  |  | Currently owns a pet (n=90) | | Currently does not own a pet (n=288) | |  |  |
| Outcome | Variable | Model | Point est | (SE) | Point est | (SE) | PO *p* | age *p* |
| Disease/Disability | |  |  |  |  |  |  |  |
| Physical Wellness | SF-12 Physical Health | PO | 52.6 | (0.89) | 50.6 | (0.49) | **0.026** | N/A |
|  |  | PO, age | 51.5 | (0.97) | 50.4 | (0.62) | 0.145 | **0.018** |
| Cognitive Function | |  |  |  |  |  |  |  |
| Verbal Learning/Memory | CVLtca | PO | 55.7 | (1.39) | 49.9 | (0.78) | **0.0002** | N/A |
|  |  | PO, age | 53.2 | (1.44) | 50.5 | (0.93) | **0.041** | **< 0.0001** |
| Visual Perception | DSS Tot | PO | 44.1 | (1.21) | 38.9 | (0.68) | **< 0.0001** | N/A |
|  |  | PO, age | 43.1 | (1.20) | 41.3 | (0.78) | 0.077 | **< 0.0001** |
| Physical Function | |  |  |  |  |  |  |  |
| Rapid Gait Speed (M/sec) | RGSpeed | PO | 1.7 | (0.04) | 1.6 | (0.02) | **0.0002** | N/A |
|  |  | PO, age | 1.7 | (0.04) | 1.6 | (0.02) | 0.139 | **< 0.0001** |
| Daily Energy Expenditure (Kcal) | TotKCal | PO | 4798.0 | (385.43) | 3184.2 | (215.06) | **0.0002** | N/A |
|  |  | PO, age | 4528.6 | (379.89) | 3862.9 | (245.93) | 0.054 | **< 0.0001** |
| Psychological Adaptation | |  |  |  |  |  |  |  |
| Psychological Wellbeing | SF-12 Mental Health | PO | 55.6 | (0.57) | 56.1 | (0.31) | 0.198 | N/A |
|  |  | PO, age | 55.6 | (0.62) | 55.8 | (0.40) | 0.400 | 0.107 |
| Depression | CESD | PO | 5.5 | (0.58) | 5.5 | (0.32) | 0.494 | N/A |
|  |  | PO, age | 6.1 | (0.62) | 5.8 | (0.40) | 0.290 | **0.023** |
| Anxiety | Perceived Stress Scale | PO | 0.8 | (0.06) | 0.7 | (0.03) | 0.099 | N/A |
|  |  | PO, age | 0.8 | (0.06) | 0.7 | (0.04) | 0.277 | 0.106 |
| Happiness | PSY01 | PO | 8.6 | (0.15) | 8.5 | (0.08) | 0.261 | N/A |
|  |  | PO, age | 8.4 | (0.16) | 8.3 | (0.10) | 0.212 | **0.002** |
| Age = age decade; PO = current pet ownership; Physical Wellbeing = Short Form-12 Physical Component Score (SF-12 PCS); Verbal Learning/Memory = California Verbal Learning Test total correct answers; Visual Perception = Weschler Adult Intelligence Scale- Revised Digit Symbol Substitution Test total score; Psychological Wellbeing = Short Form-12 Mental Component Score (SF-12 MCS); Depression = Center for Epidemiologic Studies Depression Scale (CES-D) score; Anxiety =. Perceived Stress Scale total score; Happiness = single item (1-10); est = estimate. *p*’s are 1-tailed, bold indicates *p* < .05. | | | | | | | | |

| **Supplementary Table 2**. Summary of Contribution of Current Dog Ownership (DO) to Measures of Successful Aging in Bivariate Analysis and Controlling for Age | | | | | | | | | |
| --- | --- | --- | --- | --- | --- | --- | --- | --- | --- |
|  |  |  | **Currently owns a dog** (n=52) | | Currently does not own a dog (n=325) | | |  |  |
| Outcome | Variable | Model | Point est | (SE) | Point est | | (SE) | DO *p* | age *p* |
| Disease/Disability | |  |  |  | |  |  |  |  |
| Physical Wellness | SF-12 Physical Health | DO | 52.5 | (1.19) | | 50.8 | (0.46) | 0.100 | N/A |
|  |  | DO, age | 51.2 | (1.24) | | 50.6 | (0.60) | 0.316 | **0.011** |
| Cognitive Function | |  |  |  | |  |  |  |  |
| Verbal Learning/Memory | CVLtca | DO | 56.6 | (1.82) | | 50.4 | (0.74) | **0.0009** | N/A |
|  |  | DO, age | 53.5 | (1.82) | | 50.7 | (0.90) | 0.077 | **< 0.0001** |
| Visual Perception | DSS Tot | DO | 43.8 | (1.61) | | 39.6 | (0.65) | **0.008** | N/A |
|  |  | DO, age | 41.9 | (1.52) | | 41.7 | (0.75) | 0.450 | **< 0.0001** |
| Physical Function | |  |  |  | |  |  |  |  |
| Rapid Gait Speed (M/sec) | RGSpeed | DO | 1.8 | (0.05) | | 1.6 | (0.02) | **0.0002** | N/A |
|  |  | DO, age | 1.7 | (0.05) | | 1.6 | (0.02) | 0.097 | **< 0.0001** |
| Daily Energy Expenditure (Kcal) | TotKCal | DO | 5485.2 | (500.02) | | 3256.0 | (201.46) | **< 0.0001** | N/A |
|  |  | DO, age | 4927.2 | (478.06) | | 3867.4 | (235.03) | **0.018** | **< 0 .0001** |
| Psychological Adaptation | |  |  |  | |  |  |  |  |
| Psychological Wellbeing | SF-12 Mental Health | DO | 56.2 | (0.76) | | 56.0 | (0.29) | 0.401 | N/A |
|  |  | DO, age | 56.2 | (0.80) | | 55.6 | (0.38) | 0.239 | 0.071 |
| Depression | CESD | DO | 4.6 | (0.75) | | 5.6 | (0.30) | 0.111 | N/A |
|  |  | DO, age | 5.3 | (0.79) | | 6.0 | (0.38) | 0.212 | **0.036** |
| Anxiety | Perceived Stress Scale | DO | 0.7 | (0.07) | | 0.7 | (0.03) | 0.449 | N/A |
|  |  | DO, age | 0.7 | (0.08) | | 0.7 | (0.04) | 0.229 | 0.052 |
| Happiness | PSY01 | DO | 8.7 | (0.19) | | 8.5 | (0.08) | 0.130 | N/A |
|  |  | DO, age | 8.5 | (0.20) | | 8.2 | (0.10) | 0.113 | **0.002** |
| Age = age; DO = current dog ownership; Physical Wellbeing = Short Form-12 Physical Component Score (SF-12 PCS); Verbal Learning/Memory = California Verbal Learning Test total correct answers; Visual Perception = Weschler Adult Intelligence Scale- Revised Digit Symbol Substitution Test total score; Psychological Wellbeing = Short Form-12 Mental Component Score (SF-12 MCS); Depression = Center for Epidemiologic Studies Depression Scale (CES-D) score; Anxiety = Perceived Stress Scale total score; Happiness = single item (1-10); est = estimate. *p*’s are 1-tailed, bold indicates *p* < .05. | | | | | | | | | |
|  |  |  |  |  |  | |  |  |  |

| **Supplementary Table 3**. Summary of Contribution of Current Cat Ownership (CO) to Measures of Successful Aging in Bivariate Analysis and Controlling for Age | | | | | | | | |
| --- | --- | --- | --- | --- | --- | --- | --- | --- |
|  |  |  | Currently owns a cat (n=47) | | Currently does not own a cat (n=330) | |  |  |
| Outcome | Variable | Model | Point est | (SE) | Point est | (SE) | CO *p* | age *p* |
| Disease/Disability | |  |  |  |  |  |  |  |
| Physical Wellness | SF-12 Physical Health | CO | 52.4 | (1.24) | 50.9 | (0.46) | 0.123 | N/A |
|  |  | CO, age | 51.3 | (1.29) | 50.6 | (0.59) | 0.300 | **0.009** |
| Cognitive Function | |  |  |  |  |  |  |  |
| Verbal Learning/Memory | CVLtca | CO | 56.6 | (1.96) | 50.6 | (0.73) | **0.002** | N/A |
|  |  | CO, age | 54.0 | (1.93) | 50.7 | (0.88) | **0.052** | **< 0.0001** |
| Visual Perception | DSS Tot | CO | 45.0 | (1.71) | 39.5 | (0.64) | **0.002** | N/A |
|  |  | CO, age | 44.1 | (1.61) | 41.4 | (0.74) | 0.052 | **< 0.0001** |
| Physical Function | |  |  |  |  |  |  |  |
| Rapid Gait Speed (M/sec) | RGSpeed | CO | 1.8 | (0.05) | 1.6 | (0.02) | **0.004** | N/A |
|  |  | CO, age | 1.7 | (0.05) | 1.6 | (0.02) | 0.159 | **< 0.0001** |
| Daily Energy Expenditure (Kcal) | TotKCal | CO | 4654.6 | (542.19) | 3415.0 | (202.96) | **0.016** | N/A |
|  |  | CO, age | 4265.2 | (507.42) | 3997.0 | (233.60) | 0.305 | **< 0.0001** |
| Psychological Adaptation | |  |  |  |  |  |  |  |
| Psychological Wellbeing | SF-12 Mental Health | CO | 55.5 | (0.79) | 56.1 | (0.29) | 0.235 | N/A |
|  |  | CO, age | 55.5 | (0.83) | 55.8 | (0.38) | 0.361 | 0.098 |
| Depression | CESD | CO | 6.5 | (0.80) | 5.3 | (0.30) | 0.096 | N/A |
|  |  | CO, age | 7.2 | (0.84) | 5.7 | (0.38) | **0.042** | **0.015** |
| Anxiety | Perceived Stress Scale | CO | 0.8 | (0.08) | 0.7 | (0.03) | 0.082 | N/A |
|  |  | CO, age | 0.8 | (0.08) | 0.7 | (0.04) | 0.174 | 0.097 |
| Happiness | PSY01 | CO | 8.4 | (0.20) | 8.5 | (0.08) | 0.340 | N/A |
|  |  | CO, age | 8.2 | (0.21) | 8.3 | (0.10) | 0.344 | **0.002** |
| Age = age decade; CO = current cat ownership; Physical Wellbeing = Short Form-12 Physical Component Score (SF-12 PCS); Verbal Learning/Memory = California Verbal Learning Test total correct answers; Visual Perception = Weschler Adult Intelligence Scale- Revised Digit Symbol Substitution Test total score; Psychological Wellbeing = Short Form-12 Mental Component Score (SF-12 MCS); Depression = Center for Epidemiologic Studies Depression Scale (CES-D) score; Anxiety = Perceived Stress Scale total score; Happiness = single item (1-10); est = estimate. *p*’s are 1-tailed, bold indicates *p* < .05. | | | | | | | | |
|  |  |  |  |  |  |  |  |  |

| **Supplementary Table 4**. Summary of Contribution of Pet Ownership within Last 10-years (PO10) to Measures of Successful Aging in Bivariate Analysis and Controlling for Age | | | | | | | | |
| --- | --- | --- | --- | --- | --- | --- | --- | --- |
|  |  |  | Owned a pet in the past 10-years (n=142) | | Did not own a pet in the past 10-years (n=236) | |  |  |
| Outcome | Variable | Model | Point est | (SE) | Point est | (SE) | PO10 *p* | age *p* |
| Disease/Disability | |  |  |  |  |  |  |  |
| Physical Wellness | SF-12 Physical Health | PO10 | 52.1 | -0.7 | 50.4 | -0.54 | **0.025** | N/A |
|  |  | PO10, age | 51.3 | -0.79 | 50.3 | -0.67 | 0.125 | **0.017** |
| Cognitive Function | |  |  |  |  |  |  |  |
| Verbal Learning/Memory | CVLtca | PO10 | 54.4 | -1.11 | 49.4 | -0.86 | **0.0002** | N/A |
|  |  | PO10, age | 52.7 | -1.19 | 50.2 | -1 | **0.035** | **< 0.0001** |
| Visual Perception | DSS Tot | PO10 | 42.4 | -0.97 | 38.8 | -0.76 | **0.002** | N/A |
|  |  | PO10, age | 42.3 | -0.99 | 41.4 | -0.84 | 0.206 | **< 0.0001** |
| Physical Function | |  |  |  |  |  |  |  |
| Rapid Gait Speed (M/sec) | RGSpeed | PO10 | 1.7 | -0.03 | 1.6 | -0.02 | **0.0001** | N/A |
|  |  | PO10, age | 1.7 | -0.03 | 1.6 | -0.03 | 0.068 | **< 0.0001** |
| Daily Energy Expenditure (Kcal) | TotKCal | PO10 | 4230.1 | -309.51 | 3170.8 | -239.39 | **0.004** | N/A |
|  |  | PO10, age | 4191.1 | -313.59 | 3930.1 | -264.53 | 0.236 | **< 0.0001** |
| Psychological Adaptation | |  |  |  |  |  |  |  |
| Psychological Wellbeing | SF-12 Mental Health | PO10 | 55.9 | -0.45 | 56.0 | -0.35 | 0.403 | N/A |
|  |  | PO10, age | 55.9 | -0.51 | 55.7 | -0.43 | 0.376 | 0.0828 |
| Depression | CESD | PO10 | 5.5 | -0.46 | 5.5 | -0.35 | 0.471 | N/A |
|  |  | PO10, age | 6.1 | -0.51 | 5.7 | -0.43 | 0.277 | **0.022** |
| Anxiety | Perceived Stress Scale | PO10 | 0.8 | -0.05 | 0.7 | -0.04 | 0.056 | N/A |
|  |  | PO10, age | 0.8 | -0.05 | 0.7 | -0.04 | 0.171 | 0.119 |
| Happiness | PSY01 | PO10 | 8.6 | -0.12 | 8.5 | -0.09 | 0.177 | N/A |
|  |  | PO10, age | 8.4 | -0.13 | 8.2 | -0.11 | 0.142 | **0.002** |
| Age = age decade; PO10 = pet ownership within the last decade; Physical Wellbeing = Short Form-12 Physical Component Score (SF-12 PCS); Verbal Learning/Memory = California Verbal Learning Test total correct answers; Visual Perception = Weschler Adult Intelligence Scale- Revised Digit Symbol Substitution Test total score; Psychological Wellbeing = Short Form-12 Mental Component Score (SF-12 MCS); Depression = Center for Epidemiologic Studies Depression Scale (CES-D) score; Anxiety = Perceived Stress Scale total score; Happiness = single item (1-10); est = estimate. *p*’s *are 1-tailed*, bold indicates *p* < .05. | | | | | | | | |
|  |  |  |  |  |  |  |  |  |
|  |  |  |  |  |  |  |  |  |

| **Supplementary Table 5.** Summary of Contribution of Regular Contact (PC) with a Pet (Including Pet Ownership within Last 10-Years) to Measures of Successful Aging in Bivariate Regression and Controlling for Age | | | | | | | | |
| --- | --- | --- | --- | --- | --- | --- | --- | --- |
|  |  |  | Owned pet in past 10-years or had regular contact (n=223) | | No Pet contact (n=155) | |  |  |
| Outcome |  |  | Point est | (SE) | Point est | (SE) | PC *p* | age *p* |
| **Disease/Disability** |  |  |  |  |  |  |  |  |
| Physical Wellbeing | SF-12 Physical Health | PC | 51.6 | -0.56 | 50.3 | -0.67 | 0.074 | N/A |
|  |  | PC, age | 51.0 | -0.68 | 50.3 | -0.75 | 0.190 | **0.010** |
| **Cognitive Function** |  |  |  |  |  |  |  |  |
| Verbal Learning/Memory | CVLtca | PC | 53.3 | -0.89 | 48.5 | -1.06 | **0.0003** | N/A |
|  |  | PC, age | 52.6 | -1.01 | 49.2 | -1.13 | **0.006** | **< 0.0001** |
| Visual Perception | DSS Tot | PC | 41.9 | -0.78 | 37.8 | -0.93 | **0.0005** | N/A |
|  |  | PC, age | 42.9 | -0.85 | 40.2 | -0.93 | **0.006** | **< 0.0001** |
| **Physical Function** |  |  |  |  |  |  |  |  |
| Rapid Gait Speed (M/sec) | RGSpeed | PC | 1.7 | -0.02 | 1.6 | -0.03 | **0.004** | N/A |
|  |  | PC, age | 1.6 | -0.03 | 1.6 | -0.03 | 0.108 | 0.108 |
| Daily Energy Expenditure (Kcal) | TotKCal | PC | 3734.5 | -248.8 | 3327.3 | -298.08 | 0.148 | N/A |
|  |  | PC, age | 4048.8 | -269.15 | 4011.1 | -299.01 | 0.457 | **< 0.0001** |
| **Psychological Adaptation** | |  |  |  |  |  |  |  |
| Psychological Wellbeing | SF-12 Mental Health | PC | 55.7 | -0.36 | 56.5 | -0.43 | 0.070 | N/A |
|  |  | PC, age | 55.5 | -0.44 | 56.1 | -0.48 | 0.118 | 0.112 |
| Depression | CESD | PC | 5.4 | -0.37 | 5.7 | -0.44 | 0.31 | N/A |
|  |  | PC, age | 5.8 | -0.44 | 5.9 | -0.49 | 0.422 | **0.028** |
| Anxiety | Perceived Stress Scale | PC | 0.7 | -0.04 | 0.7 | -0.04 | 0.065 | N/A |
|  |  | PC, age | 0.8 | -0.04 | 0.7 | -0.05 | 0.12 | 0.091 |
| Happiness | PSY01 | PC | 8.6 | -0.09 | 8.4 | -0.11 | 0.0885 | N/A |
|  |  | PC, age | 8.4 | -0.11 | 8.2 | -0.12 | 0.099 | **0.002** |
| Age = age decade; PC = pet ownership within the past 10 years or regular contact with a pet; Physical Wellbeing = Short Form-12 Physical Component Score (SF-12 PCS); Verbal Learning/Memory = California Verbal Learning Test total correct answers; Visual Perception = Weschler Adult Intelligence Scale- Revised Digit Symbol Substitution Test total score; Psychological Wellbeing = Short Form-12 Mental Component Score (SF-12 MCS); Depression = Center for Epidemiologic Studies Depression Scale (CES-D) score; Anxiety = Perceived Stress Scale total score; Happiness = single item (1-10); est = estimate. *p*’s *are 1-tailed*, bold indicates *p* < .05. | | | | | | | | |
|  |  |  |  |  |  |  |  |  |
|  |  |  |  |  |  |  |  |  |

| **Supplementary Table 6.** Summary of Contribution Dog Ownership within Last 10-years (DO) to Measures of Successful Aging in Bivariate Analysis and Controlling for Age | | | | | | | | |
| --- | --- | --- | --- | --- | --- | --- | --- | --- |
|  |  |  |  |  |  |  |  |  |
|  |  |  | **Dog owners in past 10-years (**n=**91)** | | **Those who did not own a dog in past 10-years (**n=**287)** | |  |  |
| **Outcome** | **Variable** | **Model** | **Point est** | **(SE)** | **Point est** | **(SE)** | **DO** *p* | **age** *p* |
| **Disease/Disability** |  |  |  |  |  |  |  |  |
| Physical Wellbeing | SF-12 Physical Health | DO | 51.9 | -0.89 | 50.8 | -0.49 | 0.139 | N/A |
|  |  | DO, age | 50.9 | -0.95 | 50.6 | -0.63 | 0.386 | **0.009** |
| **Cognitive Function** |  |  |  |  |  |  |  |  |
| Verbal Learning/Memory | CVLtca | DO | 54.7 | -1.4 | 50.2 | -0.79 | **0.002** | N/A |
|  |  | DO, age | 52.4 | -1.41 | 50.7 | -0.95 | 0.136 | **< 0.0001** |
| Visual Perception | DSS Tot | DO | 42.4 | -1.22 | 39.5 | -0.69 | **0.020** | N/A |
|  |  | DO, age | 41.4 | -1.18 | 41.9 | -0.79 | 0.357 | **< 0.0001** |
| **Physical Function** |  |  |  |  |  |  |  |  |
| Rapid Gait Speed (M/sec) | RGSpeed | DO | 1.8 | -0.04 | 1.6 | -0.02 | **< 0.0001** | N/A |
|  |  | DO, age | 1.7 | -0.04 | 1.6 | -0.02 | **0.035** | **< 0.0001** |
| Daily Energy Expenditure (Kcal) | TotKCal | DO | 4719.2 | -381.66 | 3197.5 | -216.25 | **0.0003** | N/A |
|  |  | DO, age | 4455.5 | -370.07 | 3871.9 | -249.15 | 0.077 | **< 0.0001** |
| **Psychological Adaptation** | |  |  |  |  |  |  |  |
| Psychological Wellbeing | SF-12 Mental Health | DO | 56.2 | -0.57 | 55.9 | -0.31 | 0.340 | N/A |
|  |  | DO, age | 56.2 | -0.61 | 55.6 | -0.41 | 0.155 | 0.0602 |
| Depression | CESD | DO | 5.2 | -0.57 | 5.6 | -0.32 | 0.251 | N/A |
|  |  | DO, age | 5.7 | -0.61 | 5.9 | -0.4 | 0.390 | **0.030** |
| Anxiety | Perceived Stress Scale | DO | 0.7 | -0.06 | 0.7 | -0.03 | 0.197 | N/A |
|  |  | DO, age | 0.7 | -0.06 | 0.7 | -0.04 | 0.427 | 0.085 |
| Happiness | PSY01 | DO | 8.7 | -0.14 | 8.5 | -0.08 | 0.067 | N/A |
|  |  | DO, age | 8.5 | -0.15 | 8.2 | -0.1 | **0.034** | **0.001** |
| Age = age decade; DO = dog ownership within the past 10 years; Physical Wellbeing = Short Form-12 Physical Component Score (SF-12 PCS); Verbal Learning/Memory = California Verbal Learning Test total correct answers; Visual Perception = Weschler Adult Intelligence Scale- Revised Digit Symbol Substitution Test total score; Psychological Wellbeing = Short Form-12 Mental Component Score (SF-12 MCS); Depression = Center for Epidemiologic Studies Depression Scale (CES-D) score; Anxiety = Perceived Stress Scale total score; Happiness = single item (1-10); est = estimate. *p*’s are 1-tailed, bold indicates *p* < .05. | | | | | | | | |
|  |  |  |  |  |  |  |  |  |

| **Supplementary Table 7.** Summary of Contribution of Dog Walking (DW) Among Dog Owners to Measures of Successful Aging without and with Controlling for Age | | | | | | | | |
| --- | --- | --- | --- | --- | --- | --- | --- | --- |
|  |  |  | **Walks their dogs (**n=**37)** | | **Do not walk their dogs (**n=**12)** | |  |  |
| **Outcome** | **Variable** | **Model** | **Point est** | **(SE)** | **Point est** | **(SE)** | **DW** *p* | **age** *p* |
| **Disease/Disability** |  |  |  |  |  |  |  |  |
| Physical Wellbeing | SF-12 Physical Health | DW | 52.8 | -1.37 | 52.1 | -2.2 | 0.394 | N/A |
|  |  | DW, age | 53.7 | -1.53 | 52.5 | -2.19 | 0.327 | 0.122 |
| **Cognitive Function** |  |  |  |  |  |  |  |  |
| Verbal Learning/Memory | CVLtca | DW | 57.6 | -1.84 | 57.9 | -3.24 | 0.472 | N/A |
|  |  | DW, age | 56.8 | -2.03 | 57.4 | -3.29 | 0.432 | 0.262 |
| Visual Perception | DSS Tot | DW | 43.5 | -1.68 | 45.1 | -3.23 | 0.332 | N/A |
|  |  | DW, age | 42.6 | -1.69 | 46.1 | -3.02 | 0.152 | **0.008** |
| **Physical Function** |  |  |  |  |  |  |  |  |
| Rapid Gait Speed (M/sec) | RGSpeed | DW | 1.8 | -0.06 | 1.8 | -0.1 | 0.319 | N/A |
|  |  | DW, age | 1.8 | -0.05 | 1.8 | -0.09 | 0.462 | **0.002** |
| Daily Energy Expenditure (Kcal) | TotKCal | DW | 5831.1 | -776.25 | 5578.3 | -1325.7 | 0.435 | N/A |
|  |  | DW, age | 5271.9 | -712.22 | 5764.4 | -1108.1 | 0.352 | **0.0002** |
| **Psychological Adaptation** | |  |  |  |  |  |  |  |
| Psychological Wellbeing | SF-12 Mental Health | DW | 55.8 | -0.79 | 56.4 | -1.27 | 0.334 | N/A |
|  |  | DW, age | 55.8 | -0.92 | 56.4 | -1.32 | 0.354 | 0.406 |
| Depression | CESD | DW | 4.6 | -0.65 | 4.8 | -1.15 | 0.421 | N/A |
|  |  | DW, age | 4.6 | -0.73 | 4.8 | -1.18 | 0.458 | 0.388 |
| Anxiety | Perceived Stress Scale | DW | 0.7 | -0.08 | 0.8 | -0.14 | 0.336 | N/A |
|  |  | DW, age | 0.7 | -0.09 | 0.8 | -0.14 | 0.270 | 0.178 |
| Happiness | PSY01 | DW | 8.6 | -0.22 | 9.1 | -0.37 | 0.122 | N/A |
|  |  |  | 8.5 | (0.25) | 9.1 | (0.39) | 0.100 | 0.282 |
| Age = age decade; DW = walks dog (for current dog owners only); Physical Wellbeing = Short Form-12 Physical Component Score (SF-12 PCS); Verbal Learning/Memory = California Verbal Learning Test total correct answers; Visual Perception = Weschler Adult Intelligence Scale- Revised Digit Symbol Substitution Test total score; Psychological Wellbeing = Short Form-12 Mental Component Score (SF-12 MCS); Depression = Center for Epidemiologic Studies Depression Scale (CES-D) score; Anxiety = Perceived Stress Scale total score; Happiness = single item (1-10); est = estimate; *p*’s are 1-tailed, bold indicates *p* < .05. | | | | | | | | |
